# Using Unsupervised Machine Learning as an Alternative to Curated Medical School Rankings

**DOI:** 10.1001/jamanetworkopen.2023.24100

**Published:** 2023-07-18

**Authors:** Brandon E. Turner

**Affiliations:** 1 Harvard Radiation Oncology Program, Boston, Massachusetts; 2 Department of Radiation Oncology, Dana Farber Cancer Institute, Boston, Massachusetts

## Abstract

This cross-sectional study compares an unsupervised machine learning algorithm’s ability to group schools into tiers vs the US News & World Report’s medical school rankings.

## Introduction

Annual medical school and hospital rankings, most prominently by the *US News & World Report (USNWR)*, influence prospective students’ decision-making, institutional fundraising, and hiring.^1,2^ Several stakeholders have criticized *USNWR* rankings, spurring the creation of new rankings (eg, based on research accomplishments^2^ or social mission^3^), or prompting some schools to withdraw data from ranking efforts.^4^ Rankings can provide a useful mechanism for separating schools. However, both *USNWR* and its rivals rely on editorial judgment in determining which metrics to collect and weigh to create a single ordinal ranking score despite the diversity of needs and interests among stakeholders. This study presents an alternative approach that permits stakeholders to independently determine ranking metrics and the relative importance of each metric to flexibly generate clusters (or tiers) of peer medical schools.

## Methods

This cross-sectional study followed the STROBE reporting guideline and was deemed exempt from review and the requirement for informed consent by the Mass General Brigham institutional review board because it did not involve data about human participants. We extracted data from the *USNWR* 2023 report on medical schools.^5^ Data were included from all 109 allopathic schools that received numeric ranks. We generated a set of tiers using the 8 research metrics and weights used by the *USNWR* rankings (scenario 1) (Table). We first generated clusters by applying the Ward method, an unsupervised machine learning algorithm,^6^ to a distance matrix of normalized and then weighted metric values (weights derived from *USNWR* or user-generated) (see eAppendix in Supplement 1 for details). Clusters were arranged into tiers based on the median value of the sum of weighted, normalized metrics across schools within each cluster. To examine the flexibility and performance of our tiers approach, we modified the default input to generate 2 additional sets of tiers: we added uniform random noise between −5% to 5% and between −15% to 15% to each metric (scenario 2); we added 4 metrics (using supplemental data published by *USNWR*) to the algorithm (scenario 3), namely, in-state cost of attendance, mean debt, United States Medical Licensing Examination Step 1 passing rate, and percentage of underrepresented students with minoritized race or ethnicity. We compared cluster assignments using adjusted Rand Index (ARI). All analyses were conducted using R version 4.2.1 (R Foundation for Statistical Computing) from December 2022 to February 2023.

**Table.** Metrics and Weights Used to Group Schools Into Tiers

Metrics (and prestandardized scale and units)	Weights^a^		
	Scenario 1: default *USNWR* research	Scenario 2: default *USNWR* research plus noise	Scenario 3: modified *USNWR* research
Peer research assessment (1-5)	0.15	0.15	0.15
Residency director research assessment (1-5)	0.15	0.15	0.15
Total federal research activity, $	0.30	0.30	0.30
Mean federal research activity per full-time faculty member, $	0.10	0.10	0.10
Median GPA (1.0-4.0)	0.06	0.06	0.06
Median total MCAT percentile (0-100)	0.13	0.13	0.13
Acceptance rate, %	0.01	0.01	0.01
Ratio of full-time faculty to students, log	0.10	0.10	0.10
Proportion underrepresented students with minoritized race or ethnicity, %	0	0	0.10
In-state cost of attendance, $	0	0	0.20
Mean indebtedness of 2021 graduates who incurred medical school debt, $	0	0	0.20
USMLE Step 1 passing rate, %	0	0	0.10

Abbreviations: GPA, grade point average; MCAT, Medical College Admission Test; USMLE, United States Medical Licensing Examination; *USNWR, US News & World Report*.

^a^ Weights are relative and do not necessarily add up to 1.

## Results

The total sample included all 109 allopathic schools that received numeric ranks by *USNWR*. Scenario 1 produced 8 tiers (Figure). Of the *USNWR* top 11 schools, 9 (82%) were also grouped within the 11 schools of tier 1. Of the *USNWR* top 39 schools, 38 (97%) were also grouped within the 39 schools of tiers 1 to 3. Scenario 2 showed similar clusters compared with scenario 1 even after the addition of noise to all metrics across all schools (5% noise threshold: ARI, 0.70 [95% CI, 0.67-0.72]; 15% noise threshold: ARI, 0.59 [95% CI, 0.57-0.62]) (Figure). Cluster similarity was less with scenario 3 (ARI, 0.30 [95% CI, 0.27-0.32]) after the inclusion of additional metrics (Figure).

**Figure.**
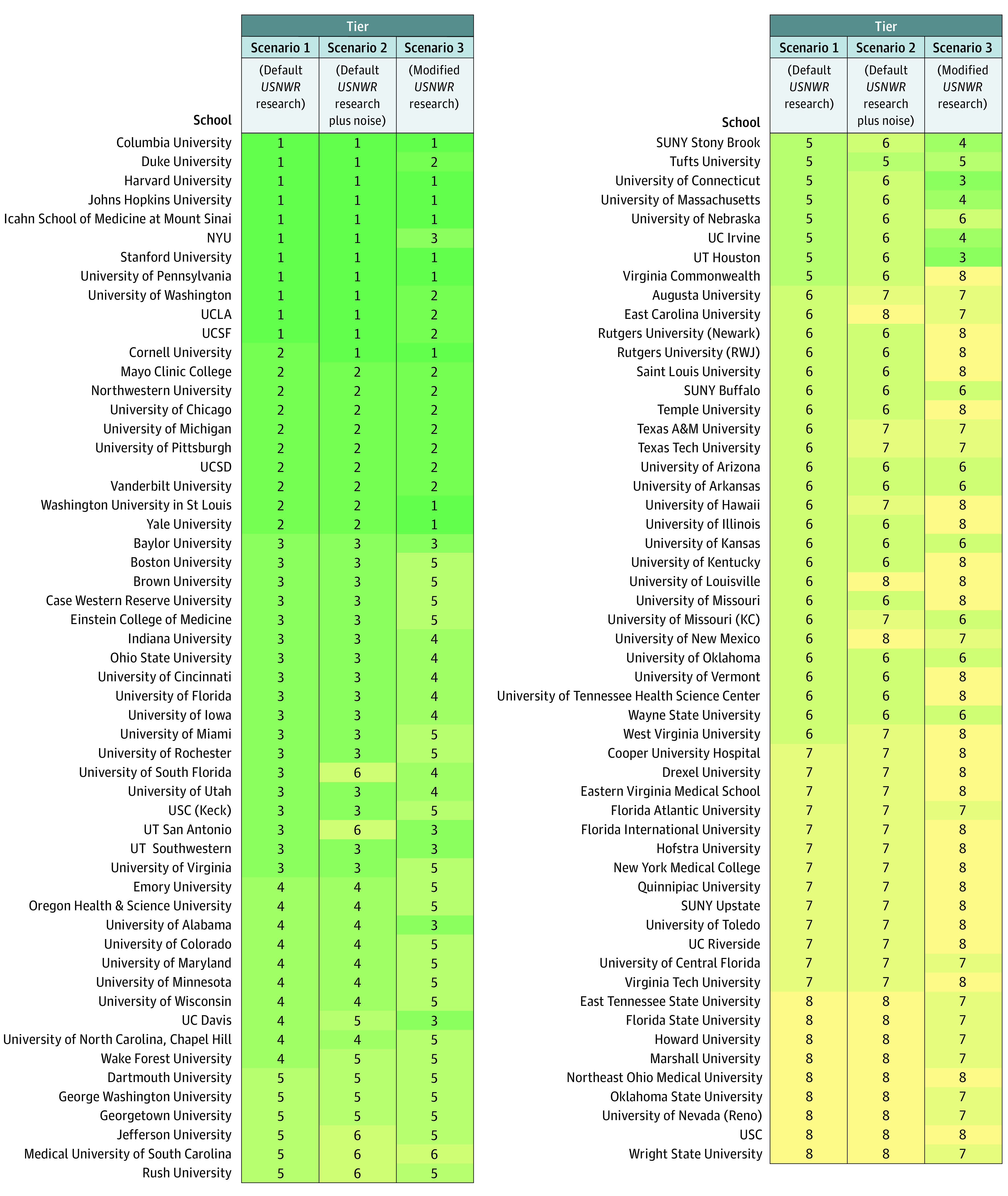
Groupings of Medical Schools Into Clusters and Tiers Using 3 Sets of Starting Metrics Each row within the grid represents the tiers for a specific school. Each column within the grid represents the results of a specific clustering scenario. Scenario 1 uses the same 8 metrics (peer research assessment, residency director research assessment, total federal research activity, mean federal research activity per full-time faculty member, median grade point average, median total Medical College Admission Test, acceptance rate, faculty-student ratio) from the *US News & World Report (USNWR)* medical school research rankings. Scenario 2 applies uniform random noise between −15% to 15% to the 8 default *USNWR* metrics before clustering. Scenario 3 includes 4 additional metrics (in-state cost of attendance, mean debt, United State Medical Licensing Examination Step 1 passing rate, and percentage of underrepresented students with minoritized race or ethnicity at the school) in addition to the 8 default *USNWR* metrics.

## Discussion

This study found that an unsupervised machine learning algorithm could group schools into tiers. The tiers were robust to random noise, suggesting that small perturbations in school metrics (such as those resulting from yearly fluctuations or differences in admissions philosophy) are less likely to dislodge schools from their peer institutions. Our tiers closely mimicked the *USNWR* rankings when the same metrics and weights were used. However, those metrics and weights represent a solitary and subjective standard. Nearly limitless combinations of metrics and weights exist which could possibly represent stakeholders’ diverse preferences. We have established an openly accessible public website (see Additional Information) where users can experiment and generate tiers using the aforementioned algorithm while selecting their preferred metrics (eg, related to research, primary care, financial aid, diversity). In this study, the addition of just 4 new metrics substantially altered cluster similarity. This study and public tool are limited by their continued reliance on data already collected by *USNWR*. However, the underlying method could be extended beyond medical schools to hospitals or other ranked entities for which one has data.

## Notes

Supplement 1. **eMethods.** Supplemental Methods
eReferences

Supplement 2.
Data Sharing Statement
