# Supplementary material for: Using Unsupervised Machine Learning as an Alternative to Curated Medical School Rankings
Source: JAMA Netw Open. 2023 Jul 18;6(7):e2324100. doi: 10.1001/jamanetworkopen.2023.24100 (PMC10354671; doi:10.1001/jamanetworkopen.2023.24100)
Supplement: Supplement 1. — eMethods. Supplemental Methods eReferences [file jamanetwopen-e2324100-s001.pdf]

Supplemental Online Content

Turner BE. Using unsupervised machine learning as an alternative to curated medical school rankings. *JAMA Netw Open.* 2023;6(7):e2324100.
doi:10.1001/jamanetworkopen.2023.24100

eAppendix. Supplemental Methods
eReferences

This supplemental material has been provided by the authors to give readers additional information about their work.

© 2023 Turner BE et al. *JAMA Network Open.*

eAppendix. Supplemental Methods

To prepare a distance matrix for clustering, we performed a series of steps:

1) We normalized the values for each metric by subtracting from each school's specific metric value the mean of that metric across all 109 schools, and then dividing the resulting value by the standard deviation for that metric across all 109 schools in our dataset (i.e. we created Z-scores).
2) We restricted outliers by setting all outliers to 1.5 times the interquartile range above or below the 3rd or 1st quartile respectively for each metric (e.g. GPA, acceptance rate, etc.).
3) We weighted each metric by multiplying each school's specific metric value by the weight value for that metric. Weight values were user-supplied or derived from USNWR methodology. The value of weights was arbitrary and served only to establish the relative importance of different metrics to each other (i.e. they do not need to sum to a specific value; weights of [0.1, 0.2, 0.3] would be equivalent to [0.2, 0.4, 0.6]). A weight of 0 would be equivalent to excluding that metric from consideration in the clustering/ranking.
4) The resulting weighted, normalized metric values from Steps 1-3 were used to calculate a pairwise Euclidean distance matrix where each school was compared to all other schools.

Details of ranking metrics can be found in the US News Methodology can be found at https://www.usnews.com/education/best-graduate-schools/articles/medical-schools-methodology. Cost and fee variables were represented using negative values (i.e. tuition of $100 was represented as -100 in the dataset prior to normalization).

Hierarchical clustering was performed using Ward's method (cluster method "ward.D2" from the **hclust** function within the *stats* package). The number of clusters could consequently be selected by determining where to "cut" the resulting dendrogram. While it is not uncommon to arbitrarily select (or use theoretical considerations to select) the number of clusters for unsupervised machine learning methods, several validity indices for determining the "optimal" number of clusters have also been proposed in an effort to simultaneously maximize the similarity within clusters and the dissimilarity between clusters.

We attempt to identify the optimal number of clusters, which will later be arranged into tiers, by combining the outcome of multiple validity index procedures using and as described in the paper for the *NbClust* package[1]. We acknowledge that the "optimal" number of clusters is a theoretical construct. For the purposes of school rankings, the number of clusters could also adequately be selected arbitrarily or for convenience.

Using *NbClust*, we first restricted the possible number of clusters to 6 to 20 for ease of analysis (i.e. setting k.min to 6 and k.max to 20). *NbClust* provides 30 indices which each represent a different procedure for determining the optimal number of clusters. These indices are described in detail in the package documentation. There is little theoretical or empirical data to draw from in identifying clusters of medical school data. Simulation studies have shown that different indices perform best for specific scenarios, but this would require prior knowledge of the "true" number of clusters in the data[2]. We thus adapted the "majority rule" approach solution by *NbClust* to take the median value from the resulting indices.

© 2023 Turner BE et al. *JAMA Network Open.*

From the 30 indices provided by *NbClust*, we used 24 indices which were consistently able to converge without error in our testing: "kl", "ch", "hartigan", "ccc", "scott", "marriot", "trcovw", "tracew", "friedman", "rubin", "cindex", "db", "silhouette", "duda", "pseudot2", "beale", "ratkowsky", "ball", "ptbiserial", "frey", "mcclain", "dunn", “sdindex", "sdbw", as described in the paper. From each index, the optimal number of clusters was calculated. We took the median value of these to determine the final cutpoint to produce clusters from our original distance matrix. If an index algorithm was not able to calculate an optimal number of clusters (e.g. did not converge), that index was omitted before calculation of the median.

In the public website we instead allow users to decide arbitrarily the number of clusters rather than trying to calculate a single optimal number. This permits a wider array of use scenarios and also facilitates server performance given the potential for large numbers of synchronous usage and the computational burden of calculating all 24 indices.

To arrange the N clusters into N tiers, we performed the following series of steps:

1. For each school in each cluster, we summed the weighted, normalized metric values which were used to generate the distance matrix in Step 4 from the previous section, thus producing a single, sum value for each school.
2. For each cluster, the median of the sums for each school in the cluster was calculated. This median value was subsequently used to represent that cluster.
3. The clusters were arranged in descending order of their representative value, with the top tier (Tier 1) corresponding to the cluster whose value was greatest. The remaining tiers were similarly assigned based on their representative value, such that the last tier (Tier N) corresponded to the cluster whose value was least.

eReferences

1. Charrad M., Ghazzali N., Boiteau V., Niknafs A. (2014). "NbClust: An R Package for Determining the Relevant Number of Clusters in a Data Set.", Journal of Statistical Software, 61(6), 1-36.
2. Milligan, G.W., Cooper, M.C. (1985). “An examination of procedures for determining the number of clusters in a data set.”, Psychometrika, 50, 159–179.

© 2023 Turner BE et al. *JAMA Network Open.*
