# Supplementary material for: Using Unsupervised Machine Learning as an Alternative to Curated Medical School Rankings
Source: JAMA Netw Open. 2023 Jul 18;6(7):e2324100. doi: 10.1001/jamanetworkopen.2023.24100 (PMC10354671; doi:10.1001/jamanetworkopen.2023.24100)
Supplement: Supplement 2. — Data Sharing Statement [file jamanetwopen-e2324100-s002.pdf]

Data Sharing Statement

Turner. Using Unsupervised Machine Learning as an Alternative to Curated Medical School Rankings. *JAMA Netw Open*. Published July 18, 2023.
doi:10.1001/jamanetworkopen.2023.24100

Data
Data available: No

Additional Information
Explanation for why data not available: Data used is proprietary (but open for purchase to the public) by US News and World Report
